# Early Death in Two Patients with Acute Promyelocytic Leukemia Presenting the bcr3 Isoform, FLT3-ITD Mutation, and Elevated WT1 Level

**DOI:** 10.1155/2013/896394

**Published:** 2013-07-07

**Authors:** Marianna Greco, Giovanni Caocci, Antonio Ledda, Adriana Vacca, Marcella Arras, Ivana Celeghini, Giorgio La Nasa

**Affiliations:** ^1^Hematology Unit and Bone Marrow Transplantation Center, “R. Binaghi” Hospital, Via Is Guadazzonis 3, 09126 Cagliari, Italy; ^2^Department of Medical Sciences, University of Cagliari, 09100 Cagliari, Italy

## Abstract

Despite major advances in the treatment of acute promyelocytic leukemia (APL), the problem of early death (ED) remains unsolved. Alongside the currently known clinical and hematological risk factors, prognostic significance has been attributed to internal tandem duplication mutations of the fms-like tyrosine kinase-3 (FLT3-ITD), hypogranular variant morphology, and the bcr-3 isoform of PML-RAR**α**. We describe premature death of two patients with the hypogranular variant of APL who presented remarkably high expression levels of Wilms' tumor gene (WT1). Our results point to WT1 as an important prognostic factor of ED that needs to be promptly evaluated in all newly diagnosed cases of APL.

## 1. Introduction

Early death (ED) in acute promyelocytic leukemia (APL) occurs in approximately 10 to 25 percent of patients within 30 days of starting induction chemotherapy and is frequently associated with severe hemorrhagic complications [1]. Although the addition of all-trans-retinoic acid (ATRA) to treatment regimens has considerably improved complete remission (CR) rates, the incidence of ED continues to be around 20% [2]. 

Elevated absolute blast and promyelocyte counts, high prothrombin time at presentation, thrombocytopenia, older age, and anemia have been reported as risk factors associated with ED in APL patients [3]. The aim of this report is to further clarify the clinical significance of molecular genetic parameters in the pathogenesis of hemorrhagic complications and ED in APL. 

The PML-RAR*α* transcript subtypes referred to as long (L or bcr-1), variant (V or bcr-2), and short (S or bcr-3), depending on the location of breakpoints within the PML site (intron 6, exon 6, and intron 3), have not been clearly associated with different prognosis or ED in APL [4].

Internal tandem duplication mutations of the fms-like tyrosine kinase-3 (FLT3-ITD) portend poor prognosis in acute leukemias and have recently been found capable of predicting ED in pediatric patients with acute APL [5]. Despite several reports of association between FLT3-ITD and other characteristics of APL, including elevated white blood cell (WBC) counts, hypogranular variant morphology (M3v), and the short (bcr-3) isoform of PML-RAR*α*, the prognostic significance of FLT3 mutations still needs to be firmly established [6].

Wilms' tumor gene 1 (WT1), located on the short arm of chromosome 11, is expressed in most patients with acute myeloid leukemia (AML) or lymphocytic leukemia (ALL) [7]. APL represents the AML subtype with the highest WT1 expression levels. A recent study found that high WT1 mRNA expression correlated with the presence of FLT3 mutations, but whether this finding had an impact on clinical cure and/or outcome was unclear [8].

## 2. Case Presentation

In the present report, we describe ED in the hypogranular variant of APL in two patients positive for the FLT3-ITD mutation presenting the bcr-3 molecular variant of the PML-RAR*α* transcript and elevated WT1 expression levels. 

The first patient, a 70-year-old female with breast cancer diagnosed in October 2009, was treated after surgical quadrantectomy with four courses of docetaxel and cyclophosphamide, followed by radiotherapy and hormonal therapy with an aromatase inhibitor. In July 2011, she complained of severe fatigue, hematuria, weight loss, and worsening of general health status. Blood tests showed leukocytosis, anemia, and thrombocytopenia. The WBC count was 11.7 × 10^9^/L, the hemoglobin (Hb) level was 8.4 mg/dL, and the platelet (PLT) count was 13 × 10^9^/L. Coagulation parameters were consistent with consumptive coagulopathy (increased D-dimer levels and hypofibrinogenemia). 

**Figure 1:**
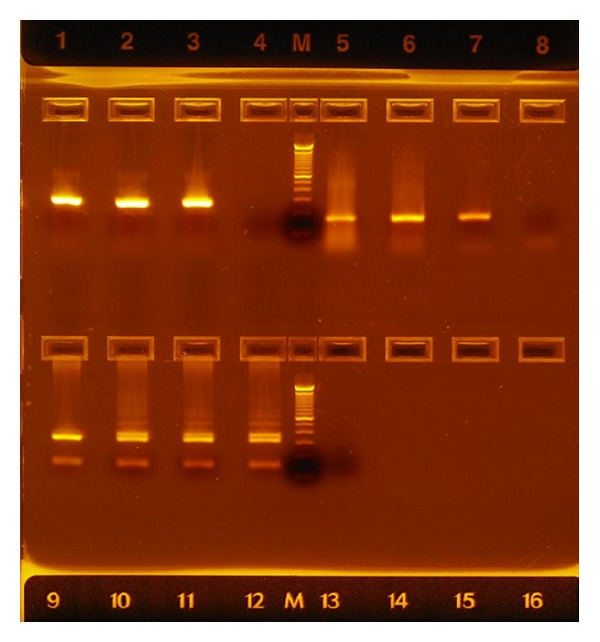
Reverse transcriptase-polymerase chain reaction (RT-PCR) and PCR for the evaluation of bone marrow samples from two APL patients. Upper panel: lanes 1 and 2: transcripts of the bcr-3 isoform in patients; lane 3: bcr-3 positive control; lane 4: no template control; M: molecular weight marker; lanes 5 and 6: primers specific for the RARA gene used for control of cDNA quality in each patient; lane 7: RARA positive control; lane 8: no template control. Lower panel: lanes 10 and 11: patients with an FLT3 ITD mutation; lane 9: normal individual; lane 12: FLT3 ITD positive control; lane 13: no template control; M: molecular weight marker.

Physical examination revealed pallor, mild hepatosplenomegaly, and petechiae. Bone marrow aspirate revealed a hypercellular marrow with 45% blast cells characterized by a reniform or bilobed nucleus and apparently agranular cytoplasm. Immunophenotyping identified the blast cells as CD45+, CD34+, CD117+, CD33+, CD13+, CD2+, CD9+, CD45RA+, HLADR−, CD66−, CD15−, CD11b−, and CD16−. Cytogenetic evaluation showed 46, XX, t(15;17) (q22;q21) in 20/20 metaphases. Qualitative polymerase chain reaction (PCR) was used to identify the presence of the PML-RAR*α* isoform bcr-3 and the FLT3-ITD mutation (Figure 1). The WT1 transcript amount, expressed as WT1 copies every 10^4^ copies of ABL, was determined by real-time quantitative PCR (RQ-PCR) performed on a 7900 real-time PCR system (Applied Biosystems, ProfileQuant Kit, Ipsogen, Marseille, France). The result obtained for our patient was 48216 copies, thereby confirming overexpression of WT1.

The patient was diagnosed with APL, classified as M3v according to the French-American-British (FAB) criteria, and admitted to our microbial-controlled ward. The patient started induction therapy with ATRA 45 mg/m^2^ for 30 days and intravenous idarubicin at a dosage of 12 mg/m^2^ on days 2, 4, 6, and 8, according to the Italian protocol AIDA (ATRA + idarubicin) [9]. Prednisone 0.5 mg/Kg was administered for ATRA syndrome prophylaxis and platelets infused with a target of at least 30 × 10^9^/L, as well as fresh frozen plasma. At day 3, the patient presented dyspnea, fever, and peripheral edema with oxygen desaturation that required treatment with a continuous positive airway pressure device and transfer to a medical intensive care unit. ATRA administration was immediately interrupted. Twenty-four hours later, the patient required intubation for worsening of pulmonary respiratory status and oxygen desaturation. The WBC count increased to 34.0 × 10^9^/L. Unfortunately, our patient died the next day. 

The second patient, a 32-year-old male, referred to our center in March 2011 for hyperpyrexia, headache, lumbar pain, weight loss, hematuria, and bleeding gums. Blood tests showed leukocytosis (WBC 30.0 × 10^9^/L), normal Hb (15.6 mg/dL), and thrombocytopenia (PLT 16 × 10^9^/L). Clot tests were slightly abnormal: only D-dimer was increased. Physical examination disclosed nothing abnormal except mild hepatosplenomegaly. A bone marrow aspirate revealed 70% hypogranular blasts with the following immunophenotype: CD45+, CD34+, CD117+, CD33+, CD13+, CD2+, CD9+, CD45RA+, HLADR−, CD66−, CD15−, CD11b−, and CD16−. Cytogenetic evaluation showed the translocation 15;17 (q22;q21). Molecular analysis revealed the bcr-3 subtype of the PML/RAR*α* fusion gene, presence of FLT3 ITD, and elevated WT1 expression (43679 copies). 

The patient was diagnosed with APL (FAB M3v) and scheduled for treatment with the AIDA protocol, steroid prophylaxis, PLT, and fresh frozen plasma transfusions. After 48 hours of treatment with ATRA and one infusion of idarubicin, the WBC count rose to 57.7 × 10^9^/L, but headache worsened, becoming unresponsive to common pain relievers. Overnight the patient started vomiting and looked confused. Cranial computed tomography (CT) scans showed multiple bilateral supratentorial intraparenchymal hemorrhage, signs of diffuse cerebral edema, and moderate mass effect with compression of the right lateral ventricle. The patient was transferred to intensive care but died the following day.

## 3. Discussion

The problem of reducing ED in APL continues to baffle researchers worldwide and has become even more prominent in view of the marked improvement of CR rates since the introduction of combination induction therapy with ATRA and other innovative drugs such as arsenic trioxide (ATO). Although it is now possible for a vast majority of patients to achieve durable CR, the risk of severe bleeding or ATRA syndrome remains a crucial aspect of APL management. The identification of risk factors associated to ED may help clinicians decide which patients need early and more intensive supportive and prophylactic care with high doses of steroids, PLT, and fresh frozen plasma transfusions and oxygen administration. Selected patients may also benefit from carefully monitored treatment with heparin or recombinant human soluble thrombomodulin [3]. 

The two clinical cases described here point to a peculiar and rapidly fatal form of APL, characterized by the contemporary presence of hypogranular morphology (M3v), short form (bcr-3) PML-RAR*α* transcripts, FLT3-ITD mutation, and elevated WT1 expression. 

Both patients presented high WBC counts at diagnosis. Investigation performed to establish whether the FAB subtype Mv3 had a higher ED rate because of hemorrhagic complications in comparison to the classical morphologic variant FAB M3 did not show any significant differences in outcomes after adjustment for WBC counts and/or relapse risk scores [10].

Some authors report that patients with the bcr-3 isoform appear to have a shorter disease-free and overall survival compared to patients with the bcr-1 isoform, independent of the initial leukocyte count [11]. Other authors associate the FLT3/IDT mutation with ED or relapse risk and suggest that patients carrying this mutation might benefit from treatment with FLT3 inhibitors in consideration of their potential ability to abrogate differentiation syndrome or coagulopathy [3, 4, 6].

Interestingly, our study indicates a possible role for WT1 overexpression as an additional risk factor for more aggressive APL. Indeed, a median value of 29590 WT1 copies has been reported in a cohort of 97 APL patients and higher values of WT1 have been associated to the presence of FLT3 mutations [8]. Both our patients had WT1 values above 40000 copies at diagnosis, suggesting that this parameter is a particularly important prognostic factor that needs to be promptly evaluated in all newly diagnosed cases of APL. However, further monitoring and study are warranted to refine the combination of risk factors associated with ED in APL.
